# Assessing the Impact of Meteorological Factors on COVID-19 Seasonality in Metropolitan Chennai, India

**DOI:** 10.3390/toxics10080440

**Published:** 2022-08-01

**Authors:** Thodhal Yoganandham Suman, Rajendiran Keerthiga, Rajan Renuka Remya, Amali Jacintha, Junho Jeon

**Affiliations:** 1 Department of Environmental Engineering, Changwon National University, Changwon 51140, Gyeongsangnam-do, Korea; sumancas2010@gmail.com; 2 School of Smart and Green Engineering, Changwon National University, Changwon 51140, Gyeongsangnam-do, Korea; 3 Ecotoxicology Division, Centre for Ocean Research, Sathyabama Institute of Science and Technology, Chennai 600119, Tamil Nadu, India; indiagermanamali@gmail.com; 4 College of Pharmaceutical Sciences, Southwest University, Chongqing 400716, China; keerthikesh@gmail.com; 5 Centre for Material Engineering and Regenerative Medicine, Bharath Institute of Higher Education and Research, Selaiyur, Chennai 600126, Tamil Nadu, India; remya.praveen5@gmail.com

**Keywords:** COVID-19, linear regression model, seasonal, temperature, wind speed, barometric pressure, PM 2.5

## Abstract

Meteorological factors may influence coronavirus disease 2019 (COVID-19) transmission. Due to the small number of time series studies, the relative importance of seasonality and meteorological factors is still being debated. From March 2020 to April 2021, we evaluated the impact of meteorological factors on the transmission of COVID-19 in Chennai, India. Understanding how the COVID-19 pandemic spreads over the year is critical to developing public health strategies. Correlation models were used to examine the influence of meteorological factors on the transmission of COVID-19. The results revealed seasonal variations in the number of COVID-19-infected people. COVID-19 transmission was greatly aggravated by temperature, wind speed, nitric oxide (NO) and barometric pressure (BP) during summer seasons, whereas wind speed and BP aggravated COVID-19 transmission during rainy seasons. Furthermore, PM 2.5, NO and BP aggravated COVID-19 transmission during winter seasons. However, their relationships fluctuated seasonally. Our research shows that seasonal influences must be considered when developing effective interventions.

## 1. Introduction

COVID-19, which developed in Wuhan, China, in December 2019, was caused by the severe acute respiratory syndrome coronavirus 2. Despite the widespread use of intervention programs, the number of people contracting COVID-19 and dying continues to rise. On 11 March 2020, the WHO designated COVID-19 a pandemic. As of 15 October 2020, there were around 39 million confirmed cases across 235 nations, regions and territories, with an estimated 1.1 million fatalities [1]. Strategic planning, crucial policies and time-worthy decisions in the global public health sector have all been impacted by the COVID-19 pandemic. Controlling COVID-19 was difficult due to the inability to fully comprehend the infectious agent and its environmental interactions. In addition, factors such as climate and atmosphere may have impacted respiratory infectious illnesses’ tendency to lead to pandemics [2,3].

The transmission of COVID infections, including the Middle East respiratory syndrome coronavirus and the severe acute respiratory syndrome (SARS), has been linked to temperature, relative humidity and wind speed [4,5]. When the warmer weather arrived in 2003, China’s Guangdong province saw a gradual drop in the incidence of severe SARS [6]. However, the epidemic was caused by additional environmental components, including the significant accumulation of air pollution. According to Cui et al. (2003), air pollution influenced the SARS pandemic that broke out in mainland China in November 2002. The study investigated the seasonal occurrence of respiratory illness epidemics, which often happen at low winter temperatures [7]. The coronavirus may survive in a subtropical environment for two weeks in low-temperature, humid locations, allowing virus transmission [8]. According to research conducted in Korea, the risk of contracting influenza increased noticeably with greater relative humidity and lower daily temperatures [9].

The number of findings on COVID-19 mortality and the connection with environmental and meteorological factors is increasing, and they are also critical. Several cited studies have been reported concerning COVID-19 mortality, infection and environmental variables. For instance, Qi et al. (2020b) discovered a strong correlation between humidity and temperature associated with COVID-19 in China [10]. Ma et al. (2020) utilized air pollution datasets and climatic factors to calculate the daily number of COVID-19 deaths in Wuhan, China, the city where the pandemic originated [2]. However, it is currently unknown how climatic conditions influence the rates of COVID-19 confirmed cases. According to the current research, a firm conclusion about the possible impact of climatic variables on COVID-19 globally has yet to be reached. Only a few studies have examined the impact of seasonality [11,12,13,14] (Table S1). In the early stages of the pandemic, most studies focused on time series with a limited time frame [15]. Meteorological influences on COVID-19 cannot be discerned from investigations conducted over a short period.

Chennai, which is one of India’s largest metropolises with around 10 million people spread across 1189 square kilometres [16]. Throughout the pandemic, Chennai has surpassed all the other districts of Tamil Nadu in the number of confirmed COVID-19 infections and fatalities per 1000 population [17]. However, we do not know how weather conditions could influence disease transmission. Therefore, using a linear regression model (LRM), we sought to determine the impact of meteorological conditions on COVID-19 confirmed cases in Chennai, India. Using the data, we may better understand the probable connections between COVID-19 and meteorological conditions. For the first time, a report from Chennai, India, on possible correlations between meteorological conditions and verified COVID-19 cases has been presented in this research.

## 2. Materials and Methods

### 2.1. Data on COVID-19 Incidence

The Directorate of Public Health and Preventive Medicine, Health and Family Welfare Department, Government of Tamil Nadu, provided COVID-19 incidence numbers for this time-series study in Chennai. In this analysis, we used daily counts of positive COVID-19 diagnoses from all reporting sources, including clinical and laboratory diagnoses. The COVID-19 database contains cases of all ages and the COVID-19 incidence data for Chennai, Tamil Nadu, between 7 March 2020, and 12 April 2021.

### 2.2. Environmental Condition Data

The mean daily temperature, wind speed, PM 2.5, NO, sulfur dioxide (SO_2_) and BP concentration were retrieved from the central pollution control board, Chennai, Tamil Nadu, India, between 1 March 2020, and 6 April 2021.

### 2.3. Data Analysis

The GraphPad Prism1 8.0 (Graphpad Inc., Harvey Motulsky, Los Angeles, CA, USA) was used for all data analysis. A descriptive analysis was first carried out to give an overall view of COVID-19 incidence and temperature, wind speed, PM 2.5, NO, SO_2_ and BP over the research period. The daily incidence of COVID-19 was matched using all independent variables. The COVID-19 incidence was then fitted to each independent variable using an LRM. The *p*-value < 0.05 was considered statistically significant in the two-sided statistical tests.

## 3. Results

### 3.1. Description of COVID-19 Outbreak, Temperature, Wind Speed, and Air Pollutants

**Figure 1:**
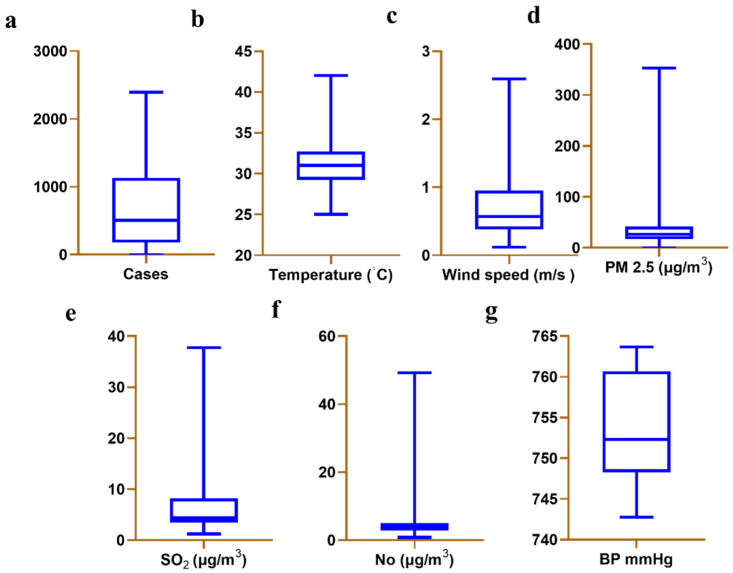
Boxplot of the weather conditions in Chennai, India from March 1, 2020, to April 12, 2021. (**a**) cases, (**b**) temperature (°C), (**c**), wind speed (m/s), (**d**) PM 2.5 (μg/m^3^), (**e**) SO_2_ (μg/m^3^), (**f**) NO (μg/m^3^), (**g**) BP (mmHg).

The statistical analysis of confirmed cases of COVID-19, temperature, wind speed, and air pollutants were shown in Figure 1 and Table 1. During the study (7 March 2020, to 12 April 2021), 264,535 confirmed cases of COVID-19 were reported in Chennai. On average, approximately 658.2 confirmed COVID-19 cases were reported per day (Figure 1a). There was a temperature range of between 25 °C and 42 °C (Figure 1b). The averages are as follows: temperature 31.07 °C (Figure 1b), wind speed 0.7279 m/s (Figure 1c), PM 2.5 32.312 µg/m^3^ (Figure 1d), SO_2_ µg/m^3^ 7.512 (Figure 1e), NO 5.697 µg/m^3^ (Figure 1f) and BP 754.0 mmHg (Figure 1g).

**Figure 2:**
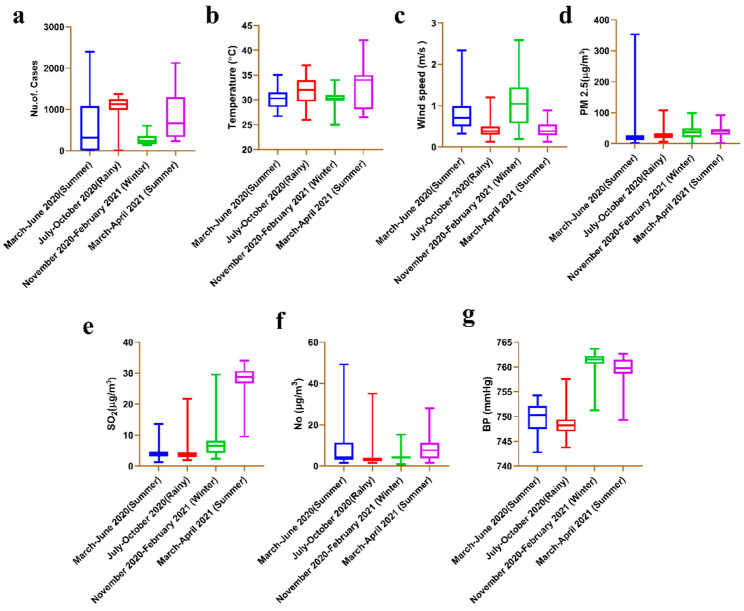
Boxplot of the summer, rainy and winter weather conditions. (**a**) cases, (**b**) temperature (°C), (**c**) wind speed (m/s), (**d**) PM 2.5 (μg/m^3^), (**e**) SO_2_ (μg/m^3^), (**f**) NO (μg/m^3^), (**g**) BP (mmHg).

Figure 2a and Table S2 show the total confirmed cases in each season from March 2020 to April 2021. The highest number of confirmed cases were reported in the period from July to October 2020 (132,289), followed by March 2020 to June 2020 (68,614), November 2020 to February 2021 (33,602) and March 2021 to April 2021 (30,570). The highest increase in confirmed cases was from July 2020 to October 2020. The average temperature was found to be highest at 32.86 °C from March 2021 to April 2021, and the lowest average was 30.19 °C from November 2020 to February 2021. From July 2020 to October 2020, the mean temperature was 32.09 °C, and from March 2020 to June 2020 it was 30.24 °C (Figure 2b and Table S3). The mean wind speed was 0.8209 m/s from March 2020 to June 2020, 0.4184 m/s from July 2020 to October 2020, 1.069 m/s from November 2020 to February 2021 and 0.4112 from March 2021 to April 2021 (Figure 2c and Table S4). The mean concentration of PM 2.5 (μg/m^3^) showed an increase of 42.10 μg m^−3^ between March 2021 and April 2021, followed by 37.66 μg/m^3^ (November 2020–February 2021), 28.60 μg/m^3^ (July 2020–October 2020) and 27.09 μg/m^3^ (March 2020–June 2020) (Figure 2d and Table S5). From March 2020 to June 2020, the SO_2_ concentration was 4.133 μg/m^3^. The mean SO_2_ concentration (μg/m^3^) in Chennai from March 2021 to April 2021 reached 28.26 μg/m^3^ (Figure 2e and Table S6). NO concentrations were 7.966 μg/m^3^ from March 2020 to June 2020, 3.784 μg/m^3^ from July 2020 to October 2020, 4.181 μg/m^3^ from November 2020 to February 2021 and 9.274 μg/m^3^ from March 2021 to April 2021 (Figure 2f and Table S7). BP mmHg readings were 749.8 mmHg from March 2020 to June 2020, 749.0 mmHg from July 2020 to October 2020, 761.34 mmHg from November 2020 to February 2021 and 759.3 mmHg from March 2021 to April 2021 (Figure 2g and Table S8).

### 3.2. Association between All Variables and COVID-19 Confirmed Cases

**Figure 3:**
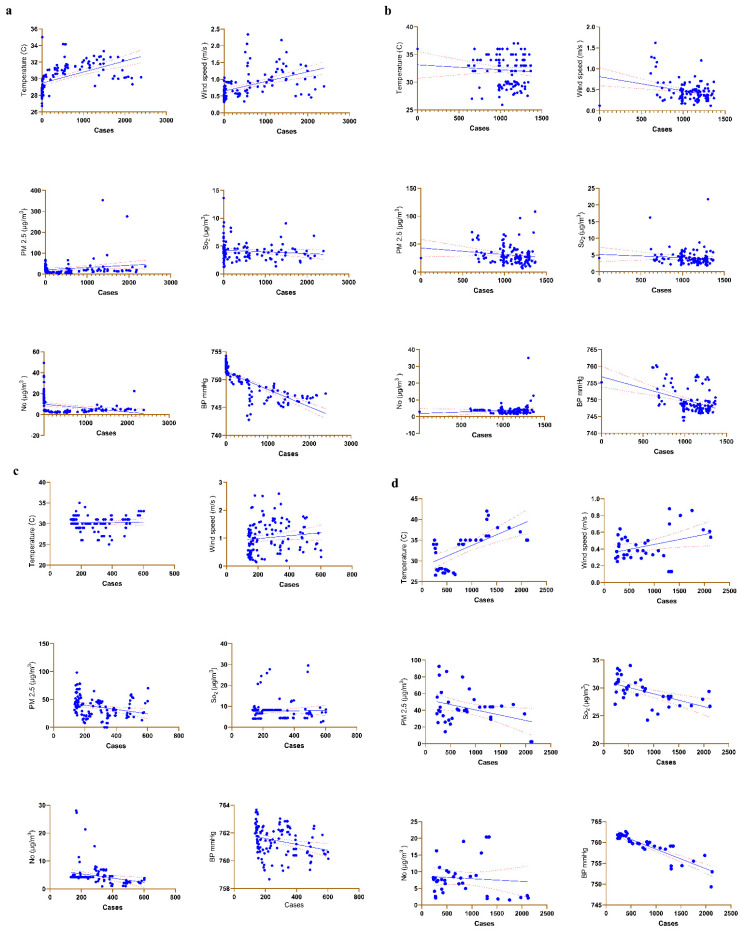
The correlation between daily COVID-19 incidence and meteorological factors (**a**) summer—1 March 2020, to 30 June 2020, (**b**) rainy season—1 July 2020, to 31 October 2020, (**c**) winter months—1 November 2020 to 28 February 2021, (**d**) summer—1 March 2021, to 6 April 2021. Blue dot represents data points, Blue line represent line of regression, Red dashed lines represent the confidence interval.

The correlation between COVID-19 transmission and meteorological conditions was studied (Figure 3). From 7 March 2020, to 6 July 2020, the frequency of new daily COVID-19 cases exhibited no significant association with PM 2.5 or SO_2_. The data showed that temperature (R^2^ = 0.2691, *p* < 0.0001), wind speed (R^2^ = 0.2278, *p* < 0.0001), NO (R^2^ = 0.08785, *p* < 0.0009) and BP (R^2^ = 0.6027, *p* < 0.0001) was significantly associated with daily COVID-19 incidence which indicated its importance in the role of COVID-19 transmission during summer (1 March 2020, to 30 June 2020) (Figure 3a and Table 2). Interestingly, during the rainy season (1 June 2020, to 31 October 2020), wind speed (R^2^ = 0.04867, *p* < 0.05) and BP (R^2^ = 0.1648, *p* < 0.001) showed significant association with daily COVID-19 incidence (Figure 3b and Table 2). However, temperature (R^2^ = 0.01552, *p* > 0.05), PM 2.5 (R^2^ = 0.007495, *p* > 0.05) and SO_2_ (R^2^ = 0.00005, *p* > 0.05) did not show a statistical significance. In the winter months (1 November 2020, to 28 February 2021), we examined the association between COVID-19 incidence and meteorological characteristics. Among the six parameters, PM 2.5 (R^2^ = 0.07301, *p* < 0.01), NO (R^2^ = 0.06935, *p* < 0.01) and BP (R^2^ = 0.07248, *p* < 0.01) were predominantly correlated with COVID-19 incidence (Figure 3c and Table 2). During the summer (1 March 2021, to 6 April 2021) temperature (R^2^ = 0.4272, *p* < 0.0001), wind speed (R^2^ = 0.1285, *p* < 0.001), PM 2.5 (R^2^ = 0.1272, *p* < 0.05), SO_2_ (R^2^ = 0.3177, *p* < 0.001) and BP (R^2^ = 0.5915, *p* < 0.001) were significantly associated with COVID-19 incidence (Figure 3d and Table 2).

## 4. Discussion

### 4.1. Pollutant Levels during COVID-19 Pandemic

Viral survival and spread can be affected by changes in temperature. Viruses are more active in cold weather, and their activity decreases as the temperature rises because the lipid layer of the virus is damaged by higher temperatures [8]. From March 2021 to April 2021, our study recorded the highest mean temperature (32.86 °C). There is a general relationship between wind speed and the amount of air pollution that can be dispersed. The present study observed high wind speeds (1.069 ms^−1^) from November 2020 to February 2021. According to Li et al. (2012), a wind speed of roughly 1.0 m s^−1^ is sufficient to disperse pollutants in a roadway canyon [18]. In our study, the average concentration of PM 2.5 μg/m^−3^ was highest in April and March 2021 (40.17 μg/m^3^) and from November 2020 to February 2021 (37.66 μg/m^3^). This shows that the concentration of PM 2.5 exceeds the WHO limit of 25 μg/m^3^ [19]. The increases in PM 2.5 concentrations recorded in Chennai might be linked to meteorological factors, mainly rainfall. Due to a lack of rain, the study period was deemed a dry season, meaning that pollutants such as PM 2.5 cannot be washed away. Soil, dust, construction, industrial emissions, traffic and biomass burning are all possible sources of PM 2.5 in Chennai [20]. From March 2021 to April 2021, SO_2_ levels were reported to be above the WHO guideline of 20 μg/m^3^ [19]. SO_2_ emissions may be caused by chemical manufacture, community power generation, petroleum refineries, transportation and metal industries [21]. In the present study, high mean NO emissions occurred in summer (March 2021 to April 2021 and March 2020 to June 2020). The low NO emission levels were observed during the rainy and winter season. In the summer, air conditioner use resulted in a significant increase in electricity consumption. A considerable quantity of fuel is used for this equipment to function, resulting in an increase in the amount of NO emissions being released [22]. The levels of BP were found above the standard limit of 760 mm-Hg from November 2020 to February 2021 [23].

### 4.2. The Association between Meteorological Factors and Daily COVID-19 Cases

The environmental conditions of 6 days prior to case confirmation were correlated because on average, symptoms showed up in the newly infected person about 5.6 days after contact [24]. False-positive tests, in general, refer to an incorrect signal of the presence of a specific illness. Poor rRT-PCR laboratory practices raise the possibility of a test result being falsely positive and must be considered. False-positive testing is crucial to managing local disease outbreaks successfully. There are many cases where people had positive test results for the coronavirus test, but it turned out that they did not have COVID-19 [25]. The effect of temperature is the most studied of all-weather features [26,27]. In this study, the temperature was associated with COVID-19 incidence from 1 March 2020, to 20 June 2020 (summer season), and 1 March 2021, to 6 April 2021 (summer season). At temperatures between 23 °C and 34.5 °C, COVID-19 infections increased [28]. Thailand’s average daily temperature was shown to be correlated with the cumulative daily number of COVID-19 cases [29]. Similar to research in Singapore, the number of new cases and total cases were strongly connected with the average temperature [30]. Another study showed that the COVID-19 pandemic was directly associated with the average temperature [26].

Wind speed does not appear to impact the number of COVID-19 cases [31]. Research on this subject has been limited so far. However, in our study, we analyzed wind speed. Interestingly, from 1 March 2020, to 30 June 2020 (summer season), 1 July 2020, to 31 October 2020, and 1 March 2021, to 6 April 2021 (summer season), the number of confirmed daily COVID-19 cases exhibited a significant positive association with wind speed. A study conducted in Iran found that the association is significant when the wind speeds are low enough to cause an outbreak [32]. Studies in the United States indicated wind speed did not affect viral dissemination [31]. In contrast, others found that wind speed was associated with decreasing COVID-19 infections [33].

During the flu season, the PM 2.5 concentration was strongly associated with an increased risk of influenza-like illness [34]. After attaching to PM, influenza remains airborne for a long time, allowing viruses that can be transmitted by air to be spread in a hospital setting [35]. In addition, PM has a deleterious effect on the human respiratory barrier integrity, which increases virus replication in the respiratory system [36]. According to the current study, the COVID-19 incidence from 1 November 2020, to 28 February 2021 (winter season), is strongly associated with the PM 2.5 concentration. Additionally, our data exhibited a significant correlation between the prevalence of COVID-19 and PM 2.5 from March 2021 to April 2021 (summer season). A PM 2.5 was not significantly related between 1 March 2020, and 30 June 2020 (summer season). These factors are significantly and not significantly connected with COVID-19 transmission since the present study’s data for PM 2.5 are inadequate to make any firm conclusions during summer seasons. In Italy, elevated levels of PM 2.5 were linked to an increased risk of viral transmission [37]. New COVID-19 cases in Milan, Italy, were related to the PM 2.5 concentrations [38].

However, there has been minimal investigation into SO_2_ correlations with everyday COVID-19 instances. In this investigation, from 1 March 2021, to 6 April 2021 (summer season), SO_2_ concentrations were significantly associated with daily COVID-19 instances. Between 1 March 2020, and 30 June 2020 (summer season), SO_2_ was not significantly correlated. Due to insufficient data for SO_2_ in the current investigation, there is an inability to draw any conclusive conclusions about how these variables affect COVID-19 transmission throughout the summer. Instead, increases in SO_2_ were linked to a decrease in COVID-19 cases daily [39]. Since there is not enough information to draw any firm conclusions about SO_2_ in this study, these variables are not significantly correlated to the spread of COVID-19.

Our data showed a strong correlation between COVID-19 occurrence with NO from 1 March 2020, to 30 June 2020 (summer season), and 1 July 2020, to 31 October 2020 (rainy season), despite the NO level being lower than US EPA guidelines [40]. Meanwhile, NO was significantly associated during summer (March 2021 to April 2021). The current study’s evidence for NO is insufficient to draw any definite conclusions; hence, these variables are both significantly and not significantly linked with COVID-19 transmission. However, the results revealed that BP is significantly associated with the spread of COVID-19 from 1 March 2020, to 30 June 2020 (summer season), 1 July 2020, to 31 October 2020, (rainy season), 1 November 2020, to 1 February 2021 (winter season), and 1 March 2021 to 6 April 2021 (summer season). COVID-19 spreads faster in places with higher atmospheric pressure than in locations with lower pressure, which may be because high-pressure areas tend to be wetter, making pathogens more active and invasive [41].

## 5. Conclusions

Our study examined meteorological factors’ effects on COVID-19 transmission in Chennai from 7 March 2020, to 12 April 2021. Overall, the results showed that: (1) Temperature, wind speed, NO and BP aggravated COVID-19 transmission during summer. (2) Wind speed and BP aggravated COVID-19 transmission during rainy seasons. (3) PM 2.5, NO and BP aggravated COVID-19 transmission during winter seasons. (4) COVID-19 transmission varied with seasonal changes. However, due to the relatively small area covered by our current data, we believe they may not accurately reflect the influence of temperature, wind speed, BP and PM 2.5 on the transmission of COVID-19. Therefore, based on the available information, we cannot draw any conclusions about the impact of temperature, wind speed, BP and PM 2.5 on the spread of COVID-19. To get into further detail in this study, more research is needed. There are several limitations to the present investigation. The first issue is that only one city is registered, which might cause specific findings to deviate from the precise impact of climatic factors on COVID-19 transmission. To confirm the outcomes of the present study, we will collect more data from other cities, nations, and regions in future studies.

## Figures and Tables

**Table 1:** Summary of COVID-19 positive cases, temperature, wind speed and air pollutants.

	Cases	Temperature	Wind Speed	PM 2.5	SO_2_	NO	Bar Pressure
number of values	402	402	402	402	402	402	402
minimum	0.000	25.00	0.1200	0.000	1.260	0.8900	742.8
25% percentile	176.0	29.25	0.3800	17.23	3.508	2.838	748.3
median	509.5	31.00	0.5700	26.34	4.370	4.155	752.4
75% percentile	1131	32.72	0.9525	42.30	8.190	5.075	760.8
maximum	2393	42.00	2.590	353.2	34.02	49.27	763.7
range	2393	17.00	2.470	353.2	32.76	48.38	20.89
mean	658.0	31.07	0.7279	32.31	7.512	5.697	754.0
std. deviation	534.9	2.591	0.4745	27.45	7.834	5.936	6.200
std. error of mean	26.68	0.1292	0.02367	1.369	0.3907	0.2961	0.3092
sum	264,535						

**Table 2:** The correlation between COVID-19 incidence and meteorological parameters, March 2020 to April 2021, in Chennai, India. The data marked with *, **, ***, **** indicated statistical significance, where *p* < 0.05, 0.0028 and 0.0009 and 0.0001 respectively.

	1 March 2020, to 30 June 2020		1 July 2020, to 31 October 2020		1 November 2020, to 28 February 2021		1 March 2021, to 6 April 2021	
Variables	Slope	R^2^	Slope	R^2^	Slope	R^2^	Slope	R^2^
temperature	0.0002010 ****	0.2691	0.001126	0.005129	0.001151	0.001794	0.001004 ****	0.4272
wind speed	0.00004836 ****	0.2278	0.00009834 ***	0.02256	0.0003886	0.01517	0.00005209 *	0.1285
PM 2.5	0.005438	0.03118	0.007116	0.007495	0.01263 **	0.07301	0.005601 *	0.1272
SO_2_	0.0002346	0.01443	0.0009985	0.007495	0.003445	0.0006598	0.0005683 ***	0.3177
NO	0.001098 ***	0.08785	0.001365	0.01255	0.002596 **	0.06935	0.001646	0.006817
BP	0.00023112 ****	0.6027	0.001412 ****	0.1648	0.0006967 **	0.07248	0.0004261 ****	0.7758

## Data Availability

The datasets supporting the conclusions of this article are included within the article.

## Supplementary Materials

The following supporting information can be downloaded at: https://www.mdpi.com/article/10.3390/toxics10080440/s1, Table S1: Summary of recent seasonal studies related to COVID-19. Table S2: An overview of cases between Summer-March 2020–June 2020, Rainy-July 2020–October 2020, (c) Winter-November 2020–February 2021, (d) Summer-March 2020–April 2021 in Chennai, India. Table S3: An overview of Temperature (°C) between Summer-March 2020–June 2020, Rainy-July 2020–October 2020, (c) Winter-November 2020–February 2021, (d) Summer-March 2020–April 2021 in Chennai, India. Table S4: An overview of wind speed (m/s) between Summer-March 2020–June 2020, Rainy-July 2020–October 2020, (c) Winter-November 2020–February 2021, (d) Summer-March 2020–April 2021 in Chennai, India. Table S5: An overview of PM 2.5 (µg/m³) between Summer-March 2020–June 2020, Rainy-July 2020–October 2020, (c) Winter-November 2020–February 2021, (d) Summer-March 2020–April 2021 in Chennai, India. Table S6: An overview of SO2 (µg/m³) between Summer-March 2020–June 2020, Rainy-July 2020–October 2020, (c) Winter-November 2020–February 2021, (d) Summer-March 2020–April 2021 in Chennai, India. Table S7: An overview of NO (µg/m³) between Summer-March 2020–June 2020, Rainy-July 2020–October 2020, (c) Winter-November 2020–February 2021, (d) Summer-March 2020–April 2021 in Chennai, India. Table S8: An overview of BP (mmHg) between Summer-March 2020–June 2020, Rainy-July 2020–October 2020, (c) Winter-November 2020–February 2021, (d) Summer-March 2020–April 2021 in Chennai, India.

## References

[1] World Health Organization (2020). Coronavirus Disease (COVID-2019), Situation Report.

[2] Ma Y., Zhao Y., Liu J., He X., Wang B., Fu S., Yan J., Niu J., Zhou J., Luo B. (2020). Effects of temperature variation and humidity on the death of COVID-19 in Wuhan, China. Sci Total Environ..

[3] Wu Y., Jing W., Liu J., Ma Q., Yuan J., Wang Y., Du M., Liu M. (2020). Effects of temperature and humidity on the daily new cases and new deaths of COVID-19 in 166 countries. Sci. Total Environ..

[4] Donnelly C.A., Ghani A.C., Leung G.M., Hedley A.J., Fraser C., Riley S., Abu-Raddad L.J., Ho L.M., Thach T.Q., Chau P. (2003). Epidemiological determinants of spread of causal agent of severe acute respiratory syndrome in Hong Kong. Lancet.

[5] Nair H., Brooks W.A., Katz M., Roca A., Berkley J.A., Madhi S.A., Simmerman J.M., Gordon A., Sato M., Howie S. (2011). Global burden of respiratory infections due to seasonal influenza in young children: A systematic review and meta-analysis. Lancet.

[6] Wallis P., Nerlich B. (2005). Disease metaphors in new epidemics: The UK media framing of the 2003 SARS epidemic. Soc. Sci. Med..

[7] Mourtzoukou E.G., Falagas M.E. (2007). Exposure to cold and respiratory tract infections. Int. J. Tuberc. Lung Dis..

[8] Chan K.H., Peiris J.S.M., Lam S.Y., Poon L.L.M., Yuen K.-Y., Seto W.H. (2011). The Effects of Temperature and Relative Humidity on the Viability of the SARS Coronavirus. Adv. Virol..

[9] Park J.E., Son W.S., Ryu Y., Choi S.B., Kwon O., Ahn I. (2020). Effects of temperature, humidity, and diurnal temperature range on influenza incidence in a temperate region. Influenza Other Respir Viruses.

[10] Qi H., Xiao S., Shi R., Ward M.P., Chen Y., Tu W., Su Q., Wang W., Wang X., Zhang Z. (2020). COVID-19 transmission in Mainland China is associated with temperature and humidity: A time-series analysis. Sci. Total Environ..

[11] Yin C., Zhao W., Pereira P. (2022). Meteorological factors’ effects on COVID-19 show seasonality and spatiality in Brazil. Environ. Res..

[12] Liu X., Huang J., Li C., Zhao Y., Wang D., Huang Z., Yang K. (2021). The role of seasonality in the spread of COVID-19 pandemic. Environ. Res..

[13] Yang X.D., Li H.L., Cao Y.E. (2021). Influence of meteorological factors on the COVID-19 transmission with season and geographic location. Int. J. Environ. Res. Public Health.

[14] Nottmeyer L.N., Sera F. (2021). Influence of temperature, and of relative and absolute humidity on COVID-19 incidence in England-a multi-city time-series study. Environ. Res..

[15] Smit A.J., Fitchett J.M., Engelbrecht F.A., Scholes R.J., Dzhivhuho G., Sweijd N.A. (2020). Winter is coming: A southern hemisphere perspective of the environmental drivers of SARS-CoV-2 and the potential seasonality of COVID-19. Int. J. Environ. Res. Publ. Health.

[16] CMDA (2008). Second Master Plan for Chennai Metropolitan Area 2026 Chennai Metropolitan Development Authority.

[17] Laxminarayan R., Wahl B., Dudala S.R., Gopal K., Mohan B.C., Neelima S., Jawahar Reddy K.S., Radhakrishnan J., Lewnard J.A. (2020). Epidemiology and transmission dynamics of COVID-19 in two Indian states. Science.

[18] Li L., Yang L., Zhang L.J., Jiang Y. (2012). Numerical study on the impact of ground heating and ambient wind speed on flow fields in street canyons. Adv. Atmos. Sci..

[19] WHO (2018). Ambient (Outdoor) Air Pollution. https://www.who.int/news-room/fact-sheets/detail/ambient-(outdoor)-air-quality-and-health.

[20] Raj M.G., Karthikeyan S., Raj M.G., Karthikeyan S. (2019). Effect of modes of transportation on commuters’ exposure to fine particulate matter (PM 2.5) and nitrogen dioxide (NO2) in Chennai, India. Environ. Eng. Res..

[21] Sarkar S. (2011). Engendering Trafficking and Human Security: A Comparative Study of India and Hungary. Int. J. Dev. Res. Quant. Tech..

[22] Al-Hurban A., Khader S., Alsaber A., Pan J. (2021). Air Quality Assessment in the State of Kuwait during 2012 to 2017. Atmosphere.

[23] Atmospheric Pressure. http://www.britannica.com/EBchecked/topic/41486/atmospheric-pressure.

[24] Cucinotta D., Vanelli M. (2020). WHO declares COVID-19 a pandemic. Acta Bio Med. Atenei Parm..

[25] Roy S. (2021). Physicians’ Dilemma of False-Positive RT-PCR for COVID-19: A Case Report. SN Compr. Clin. Med..

[26] Liu J., Zhou J., Yao J., Zhang X., Li L., Xu X., He X., Wang B., Fu S., Niu T. (2020). Impact of meteorological factors on the COVID-19 transmission: A multi-city study in China. Sci. Total Environ..

[27] Tosepu R., Gunawan J., Effendy D.S., Lestari H., Bahar H., Asfian P. (2020). Correlation between weather and COVID-19 pandemic in Jakarta, Indonesia. Sci. Total Environ..

[28] Ismail I.M., Rashid M.I., Ali N., Altaf B.A.S., Munir M. (2022). Temperature, humidity and outdoor air quality indicators influence COVID-19 spread rate and mortality in major cities of Saudi Arabia. Environ. Res..

[29] Tantrakarnapa K., Bhopdhornangkul B., Nakhaapakorn K. (2020). Influencing factors of COVID-19 spreading: A case study of Thailand. J. Public Health.

[30] Pani S.K., Lin N.H., RavindraBabu S. (2020). Association of COVID-19 pandemic with meteorological parameters over Singapore. Sci. Total Environ..

[31] Bashir M.F., Ma B., Bilal Komal B., Bashir M.A., Tan D., Bashir M. (2020). Correlation between climate indicators and COVID-19 pandemic in New York, USA. Sci. Total Environ..

[32] Ahmadi M., Sharifi A., Dorosti S., Jafarzadeh Ghoushchi S., Ghanbari N. (2020). Investigation of effective climatology parameters on COVID-19 outbreak in Iran. Sci. Total Environ..

[33] Rosario D.K.A., Mutz Y.S., Bernardes P.C., Conte-Junior C.A. (2020). Relationship between COVID-19 and weather: Case study in a tropical country. Int. J. Hyg. Environ. Health.

[34] Feng S., Gao D., Liao F., Zhou F., Wang X. (2016). The health effects of ambient PM2. 5 and potential mechanisms. Ecotoxicol. Environ. Saf..

[35] Lindsley W., Blachere F.M., Thewlis R.E., Vishnu A., Davis K.A., Cao G., Palmer J.E., Clark K.E., Fisher M.A., Khakoo R. (2010). Measurements of Airborne Influenza Virus in Aerosol Particles from Human Coughs. PLoS ONE.

[36] Xian M., Ma S., Wang K., Lou H., Wang Y., Zhang L., Wang C., Akdis C.A. (2020). Particulate matter 2.5 causes deficiency in barrier integrity in human nasal epithelial cells. Allergy Asthma Immunol. Res..

[37] Lolli S., Chen Y.C., Wang S.H., Vivone G. (2020). Impact of meteorological conditions and air pollution on COVID-19 pandemic transmission in Italy. Sci. Rep..

[38] Zoran M.A., Savastru R.S., Savastru D.M., Tautan M.N. (2020). Assessing the relationship between surface levels of PM2.5 and PM10 particulate matter impact on COVID-19 in Milan, Italy. Sci. Total Environ..

[39] Sangkham S., Thongtip S., Vongruang P. (2021). Influence of air pollution and meteorological factors on the spread of COVID-19 in the Bangkok metropolitan region and air quality during the outbreak. Environ Res..

[40] United States Environmental Protection Agency (2016). NAAQS Table. https://www.epa.gov/criteria-air-pollutants/naaqs-table.

[41] Aidoo E.N., Adebanji A.O., Awashie G.E., Appiah S.K. (2021). The effects of weather on the spread of COVID-19: Evidence from Ghana. Bull. Natl. Res. Cent..

