# Supplementary material for: Assessing the Impact of Meteorological Factors on COVID-19 Seasonality in Metropolitan Chennai, India
Source: Toxics. 2022 Aug 1;10(8):440. doi: 10.3390/toxics10080440 (PMC9414974; doi:10.3390/toxics10080440)
Supplement: Supplementary file 1 [file toxics-10-00440-s001.zip › toxics-1808626-SM.pdf]

toxics

MDPI

Supplementary Materials: Assessing the Impact of Meteorological Factors on COVID-19 Seasonality in Metropolitan Chennai, India

Thodhal Yoganandham Suman, Rajendiran Keerthiga, Rajan Renuka Remya, Amali Jacintha and Junho Jeon

Table S1. Summary of recent seasonal studies related to COVID-19.

Study (Area)	Period	Pollutant type	Key observation	Reference
Brazil	February 25 to November 15, 2020	Temperature, humidity, wind speed and precipitation	Cold, dry and windless conditions aggravated COVID-19 transmission.	11
Brazil, Argentina, South Africa, Peru, Chile	March to November 2020	Metrological factors and air pollution	Cold season 8.72% increase of the total infections, warm season to a 46.38 ± 29.10% reduction	12
Wuhan, Beijing, Urumqi and Dalian	8 December 2019-24 October 2020	Temperature, relative humidity, average wind speed and total precipitation	Temperature and relative humidity were mainly the driving factors on COVID-19 transmission,	13
54 English cities	30th January to October 31, 2020.	Temperature, absolute humidity, and relative humidity	Humidity exhibited elevated risk	14

Table S2. An overview of cases between Summer -March 2020-June 2020), Rainy -July 2020-October 2020), c) Winter -November 2020-February 2021, d) Summer -March 2020-April 2021 in Chennai, India.

	March-June 2020(Summer)	July-October 2020(Rainy)	November 2020-February 2021 (Winter)	March-April 2021 (Summer)
Number of values	122	123	120	37
Minimum	0.000	0.000	134.0	229.0
25% Percentile	7.000	986.0	156.3	334.5
Median	312.5	1130	232.0	664.0
75% Percentile	1083	1250	351.5	1297
Maximum	2393	1369	603.0	2124
Range	2393	1369	469.0	1895
Mean	562.4	1076	275.5	826.2
Std. Deviation	670.6	213.3	133.1	564.9
Std. Error of Mean	60.71	19.23	12.15	92.86
Sum	68614			

Table S3. An overview of Temperature (°C) between Summer -March 2020-June 2020, Rainy -July 2020-October 2020, c) Winter - November 2020-February 2021, d) Summer - March 2020-April 2021 in Chennai, India.

	March-June 2020(Summer)	July-October 2020(Rainy)	November 2020-February 2021 (Winter)	March-April 2021 (Summer)
Number of values	116	123	120	43
Minimum	26.72	25.92	25.00	26.56
25% Percentile	28.60	29.70	30.00	28.16
Median	30.31	32.00	30.00	34.00
75% Percentile	31.48	34.00	31.00	35.00
Maximum	35.00	37.00	34.00	42.00
Range	8.280	11.08	9.000	15.44
Mean	30.24	32.09	30.19	32.86
Std. Deviation	1.770	2.642	1.657	4.128
Std. Error of Mean	0.1643	0.2382	0.1512	0.6296

Table S4. An overview of wind speed (m/s) between Summer -March 2020-June 2020, Rainy - July 2020-October 2020, c) Winter - November 2020-February 2021, d) Summer - March 2020-April 2021 in Chennai, India.

	March-June 2020(Summer)	July-October 2020(Rainy)	November 2020-February 2021 (Winter)	March-April 2021 (Summer)
Number of values	116	123	120	43
Minimum	0.3200	0.1200	0.1900	0.1300
25% Percentile	0.5000	0.3000	0.5725	0.2900
Median	0.7000	0.3800	1.040	0.3800
75% Percentile	0.9875	0.5000	1.445	0.5400
Maximum	2.340	1.200	2.590	0.8800
Range	2.020	1.080	2.400	0.7500
Mean	0.8209	0.4184	1.069	0.4112
Std. Deviation	0.4106	0.1689	0.5413	0.1904
Std. Error of Mean	0.03812	0.01523	0.04941	0.02903

Table S5. An overview of PM 2.5 (μg/m³) between Summer - March 2020-June 2020, Rainy - July 2020-October 2020, c) Winter - November 2020-February 2021, d) Summer - March 2020-April 2021 in Chennai, India.

	March-June 2020(Summer)	July-October 2020(Rainy)	November 2020-February 2021 (Winter)	March-April 2021 (Summer)
Number of values	116	123	120	43
Minimum	1.440	6.760	0.000	2.460
25% Percentile	11.11	18.14	21.25	30.32
Median	17.65	25.52	37.21	40.97
75% Percentile	27.67	33.57	48.70	46.94
Maximum	353.2	108.3	98.21	92.57
Range	351.8	101.5	98.21	90.11
Mean	27.09	28.60	37.66	42.10
Std. Deviation	41.55	15.92	19.46	19.05
Std. Error of Mean	3.858	1.435	1.776	2.906

Table S6. An overview of SO_2 (μg/m³) between Summer- March 2020-June 2020, Rainy- July 2020-October 2020, c) Winter- November 2020-February 2021, d) Summer- March 2020-April 2021 in Chennai, India.

	March-June 2020(Summer)	July-October 2020(Rainy)	November 2020-February 2021 (Winter)	March-April 2021 (Summer)
Number of values	116	123	120	43
Minimum	1.260	1.840	2.320	9.500
25% Percentile	3.058	3.030	4.350	26.78
Median	3.900	3.650	6.595	28.76
75% Percentile	4.703	4.410	8.238	30.71
Maximum	13.62	21.72	29.54	34.02
Range	12.36	19.88	27.22	24.52
Mean	4.133	4.017	6.925	28.26
Std. Deviation	1.762	2.074	3.711	4.108
Std. Error of Mean	0.1636	0.1870	0.3388	0.6265

Table S7. An overview of NO (μg/m³) between Summer- March 2020-June 2020, Rainy- July 2020-October 2020, c) Winter - November 2020-February 2021, d) Summer - March 2020-April 2021 in Chennai, India.

	March-June 2020(Summer)	July-October 2020(Rainy)	November 2020-February 2021 (Winter)	March-April 2021 (Summer)
Number of values	116	123	120	43
Minimum	1.600	1.570	0.8900	1.530
25% Percentile	3.100	2.370	3.570	3.700
Median	4.160	3.480	4.230	7.690
75% Percentile	11.38	4.110	4.468	11.28
Maximum	49.27	35.08	15.29	28.05
Range	47.67	33.51	14.40	26.52
Mean	7.966	3.784	4.181	9.274
Std. Deviation	8.637	3.259	1.768	6.921
Std. Error of Mean	0.8019	0.2938	0.1614	1.055

Table S8. An overview of BP (mmHg) between Summer- March 2020-June 2020, Rainy- July 2020-October 2020, c) Winter - November 2020-February 2021, d) Summer- March 2020-April 2021 in Chennai, India.

	March-June 2020(Summer)	July-October 2020(Rainy)	November 2020-February 2021 (Winter)	March-April 2021 (Summer)
Number of values	116	123	120	43
Minimum	742.8	743.8	751.3	749.3
25% Percentile	747.5	747.0	760.6	758.7
Median	750.3	748.2	761.5	759.8
75% Percentile	752.2	749.4	762.2	761.5
Maximum	754.3	757.6	763.7	762.6
Range	11.48	13.81	12.41	13.29
Mean	749.8	749.0	761.3	759.3
Std. Deviation	2.670	3.104	1.569	2.831
Std. Error of Mean	0.2479	0.2799	0.1432	0.4317
